# Epidemiological trends and risk factors related to lower urinary tract symptoms around childbirth: a one-year prospective study

**DOI:** 10.1186/s12889-023-17065-w

**Published:** 2023-10-31

**Authors:** Xiaojuan Wang, Hongyan Wang, Ping Xu, Minna Mao, Suwen Feng

**Affiliations:** 1 School of Medicine, Zhejiang University, No.866 Yu Hang Tang Road, Hangzhou, Zhejiang Province 310058 China; 2 Women’s Hospital, School of Medicine, Zhejiang University, No.1 Xue Shi Road, Hangzhou, Zhejiang Province 310006 China

**Keywords:** Lower urinary tract symptoms, Prevalence, Risk factors, Pregnancy, Postpartum period, Prospective studies

## Abstract

**Background:**

Lower urinary tract symptoms (LUTS) are prevalent and distressing concerns for women worldwide. The prevalence of LUTS reaches the first peak during pregnancy and postnatal period. However, less attention has been paid to LUTS around childbirth and little progress has been made in the prevention of LUTS. Understanding the epidemiological characteristics of LUTS around childbirth would inform decision making for health care providers and perinatal women in the prevention of LUTS. The study aims to investigate the epidemiological trends and associated risk factors related to LUTS around childbirth.

**Methods:**

Pregnant women were consecutively enrolled during pregnancy in the obstetrical wards of a tertiary hospital and followed up at 6–8 weeks and one year postpartum through a prospective design. Urinary incontinence was assessed with the International Consultation on Incontinence Modular Questionnaire-Urinary Incontinence Short Form. Other symptoms were measured with questions based on definitions of the International Incontinence Society. Multiple logistic regression was used to examine the risk factors for LUTS including urinary incontinence, increased daytime frequency, nocturia and urgency. The report followed the STROBE statement.

**Results:**

A total of 1243 pregnant women participated in this study. The prevalence of at least one type of storage symptoms was 94%, 55% and 35% in late pregnancy, at 6–8 weeks and one year postpartum, respectively. The prevalence of urinary incontinence remained at 21% within one year postpartum. The majority of the participants suffered from mild to moderate urinary incontinence. Age, job, BMI before pregnancy, gestational diabetes mellitus, urinary tract infection history, previous history of LUTS, age at first birth and birth mode were predictors of LUTS one year postpartum.

**Conclusion:**

LUTS were highly prevalent during pregnancy and postnatal period. The prevalence of urinary incontinence was more stable than that of other LUTS within one year postpartum. Women aged more than 35 years, engaging in manual work, with gestational diabetes mellitus, with a history of urinary tract infection and LUTS, with advanced age at first birth and vaginal delivery were more likely to suffer from LUTS postpartum. The findings provided a novel and deep insight into the epidemiological trends and related risk factors of LUTS around childbirth.

**Supplementary Information:**

The online version contains supplementary material available at 10.1186/s12889-023-17065-w.

## Background

Lower urinary tract symptoms (LUTS) are prevalent and distressing concerns for women worldwide, which could have a broad and detrimental impact on quality of life and be a substantial economic burden to the individuals and society [1–6]. LUTS consist of storage, voiding and post micturition symptoms. Among LUTS, storage symptoms, including urinary incontinence, increased daytime frequency, nocturia and urgency, are more prevalent and tend to have a greater impact on quality of life than other LUTS [7–12]. According to two large population-based studies, more than half of adult women suffered from storage symptoms [8, 11]. Besides, it is estimated that 14% of women would undergo incontinence-related surgery over their lifetime [13]. As the aging population grows, women affected by LUTS and the demand for health care will increase further over the next decades [13]. However, LUTS, especially LUTS around childbirth, did not receive sufficient attention from both the health care providers and the public [14–16].

The prevalence of LUTS reaches the first peak during pregnancy and postnatal period [17, 18]. The stretching of pelvic floor muscles, connective tissues and nerves due to pregnancy and childbirth may lead to the injury of muscles and nerves, contributing to the occurrence of LUTS [19]. However, studies regarding the prevalence of LUTS around childbirth were limited and primarily focused on urinary incontinence and primiparas. A large study conducted in Norway showed that 58% of women suffered from urinary incontinence during late pregnancy and nearly one third of primiparous women were affected by urinary incontinence at 6 months postpartum [20, 21]. Another large study of primiparas conducted in China indicated that 27% of women suffered from urinary incontinence in late pregnancy but only 7% suffered from incontinence at 6 months postpartum [22]. So far, only a few studies explored the prevalence of all storage symptoms during pregnancy and the results were inconsistent. Sun et al. reported that nocturia was the most common LUTS (60%) during pregnancy in Taiwan, followed by urinary incontinence, urgency and increased daytime frequency [23]. Similarly, Liang et al. found that 51% of primiparas suffered from nocturia during late pregnancy [24]. While Balik et al. found that increased daytime frequency was more prevalent than other LUTS during late pregnancy [25]. To date, the natural process of storage symptoms from pregnancy to postnatal period was unknown. Compared with the studies regarding LUTS in middle-aged and old women, studies exploring all storage symptoms around childbirth and enrolling both primiparas and multiparas were scarce.

LUTS can be treated and prevented [26–28]. Identifying risk factors for LUTS around childbirth remains the key for early intervention of LUTS. Multiple factors may lead to the occurrence of LUTS in women including sociodemographic factors (e.g., age), obesity, lifestyle-related factors (e.g., coffee consumption), medical history (e.g., gestational diabetes mellitus) and obstetrical factors (e.g., birth mode) [5]. However, studies regarding risk factors for LUTS around childbirth considered only some of the above factors and were limited to urinary incontinence [17, 22, 29–31]. Yet, there is a lack of studies exploring the risk factors for all storage symptoms around childbirth systematically. There is evidence that women who suffered from LUTS postpartum were much more likely to suffer from persistent and long-term LUTS [31, 32]. It was worth noting that pregnant women would see the doctor regularly for antenatal and postnatal routine examination, which provides an ideal opportunity for health care providers to perform risk assessment and intervene accordingly. Understanding the risk factors for LUTS postpartum would inform decision making for health care providers and perinatal women in the prevention of LUTS.

This study aims to investigate the prevalence and associated risk factors for storage symptoms around childbirth through a large prospective study with long-term follow-up, providing novel and valuable evidence for the early prevention of LUTS.

## Methods

### Participants

Pregnant women were consecutively enrolled in the obstetrical wards of a tertiary hospital in Hangzhou, a provincial capital city in eastern China, from January to June 2020, and were followed up at 6 to 8 weeks and one year postpartum. The inclusion criteria were as follows: (1) aged 18 years or older; (2) with a singleton and term pregnancy; (3) willing to participate in the follow-up study. Women were excluded from the study if they had: (1) active urinary tract infection; (2) stillbirth; (3) a fetus with congenital malformation; (4) severe comorbidities such as severe cardiac diseases and kidney diseases.

### Measurements

Baseline data such as sociodemographic variables and lifestyle-related variables were collected in late pregnancy with a self-designed questionnaire. Obstetrical data such as parity and birth mode were obtained from medical records after childbirth. LUTS were assessed with a validated questionnaire and standardized definitions. All data were collected by trained researchers who were not involved in the treatment or nursing of the participants. Baseline data were collected through a pencil and paper survey. Follow-up data were obtained through an electronic questionnaire based on the follow-up platform of the hospital.

### Outcome

In the study, outcome refers to storage symptoms of LUTS, consisting of urinary incontinence, increased daytime frequency, nocturia and urgency. Urinary incontinence was assessed with the International Consultation on Incontinence Modular Questionnaire-Urinary Incontinence Short Form (ICIQ-UI SF), which was widely used to evaluate the prevalence, severity and type of urinary incontinence and indicated good reliability and validity in Chinese population [33, 34]. Other LUTS were measured with questions based on definitions of the International Incontinence Society [7]. Participants were asked “How many times did you urinate by day?” Increased daytime frequency refers to voiding more than 7 times by day [11]. Nocturia was assessed with the question “How many times did you have to wake to void at night?” Participants were considered to have nocturia if they voided two or more times at night [5]. Urgency was assessed with the question “How often did you have a sudden compelling desire to urinate, which was difficult to defer?” Participants were considered to have urgency if they had the complaint of a sudden desire to urinate [7].

### Statistical analysis

Sample size was calculated based on the formula n = 1.96^2^*p*(1-*p*) (*DEFF*) /*d*^2^ [35]. In the study, the expected proportion *p* was estimated to be 30-33% according to the prevalence of urinary incontinence one year postpartum in previous studies [17, 36]. The desired absolute precision *d* was usually around ± 5% for estimated *p* in the range of 20-80%. The *DEFF* was estimated to be 2 due to non-random sampling. Therefore, the required sample size was 680. Considering the loss to follow-up, the sample size was estimated to be 816, allowing a 20% dropout rate.

Descriptive analysis was applied to describe the characteristics of participants, and the prevalence and severity of LUTS. In the study, candidate risk factors for LUTS were identified based on literature review and clinical reasoning, amongst which risk factors with *p* value less than 0.2 in univariate analysis were included for multivariate analysis. An independent *t*-test and chi-square test were performed to detect the differences between groups in risk factors for LUTS one year postpartum. Multiple logistic regression with a backward process was used to examine risk factors for LUTS including urinary incontinence, increased daytime frequency, nocturia and urgency. Statistical analysis was performed with SPSS software, version 22.0 (IBM Corp., Armonk, NY). A *p*-value less than 0.05 was considered statistically significant.

## Results

**Fig. 1:**
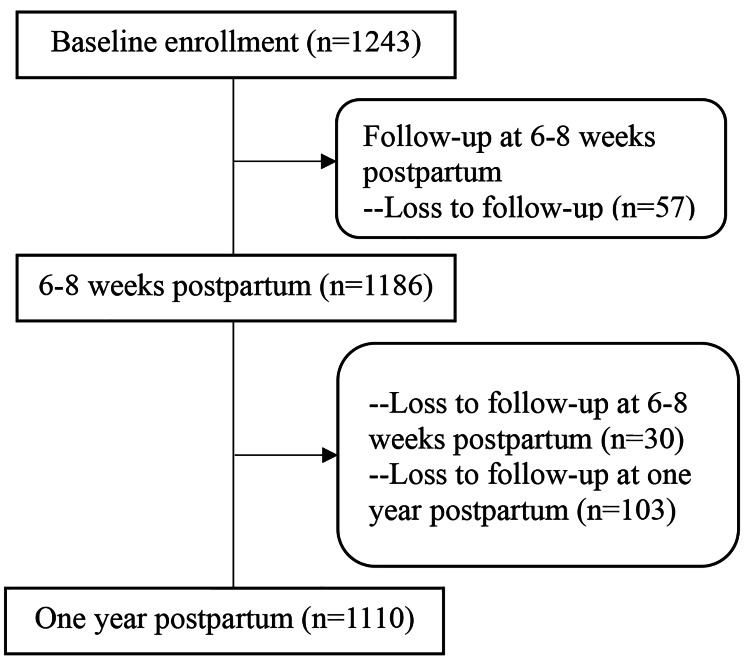
Flow chart of the follow-up study

As shown in Fig. 1, a total of 1243 pregnant women participated in this study, amongst whom 1186 (95%) and 1110 (89%) participants completed the follow-up study at 6 to 8 weeks postpartum and one year postpartum, respectively. Most participants were 35 years or younger, living in the city and engaging in mental work. The majority of pregnant women had good lifestyle. Few participants drank or smoked. As for medical history, 15% of the participants had a history of urinary tract infection and 12% of the participants had urinary incontinence before pregnancy. Approximately a quarter of the participants had gestational diabetes mellitus. About two thirds of the participants were primiparous and more than half had vaginal birth. The characteristics of participants at baseline were demonstrated in Table 1.

**Table 1:** Characteristics of participants at baseline(n = 1243)

Variables	group	n (%)/M ± SD
**Sociodemographic variables**		
Age(years)	≤ 35	1077(87)
	>35	166(13)
Place of residence	City	1000(80)
	Country	243(20)
Job	Mental work	1087(87)
	Manual work	156(13)
**BMI before pregnancy**(kg/m^2^)	—	21.1 ± 2.8
**Lifestyle-related variables**		
Smoking	No	1239(99)
	Yes	4(1)
Alcohol consumption	No	1221(98)
	Yes	22(2)
Tea consumption	< once a week	1157(93)
	≥ once a week	86(7)
Coffee consumption	< once a week	1164(94)
	≥ once a week	79(6)
Fluid consumption	<1000ml per day	500(40)
	≥ 1000ml per day	743(60)
**Medical history**		
Menstrual status	Regular	1024(82)
	Irregular	219(18)
Childhood enuresis	No	1130(91)
	Yes	113(9)
Family history of urinary incontinence	No	1187(95)
	Yes	56(5)
History of urinary tract infection	No	1052(85)
	Yes	191(15)
Constipation	No	1032(83)
	Yes	211(17)
Urinary incontinence before pregnancy	No	1088(88)
	Yes	155(12)
Gestational diabetes mellitus	No	959(77)
	Yes	284(23)
**Obstetrical variables**		
Age at first birth (years)	≤ 30	942(76)
	>30	301(24)
Prenatal BMI(kg/m^2^)	—	26.4 ± 3.0
Parity	0(primiparous)	788(63)
	≥ 1(multiparous)	455(37)
Birth mode ^a^	Cesarean section	503(41)
	Vaginal delivery	737(59)
Birth weight(g)^a^	<4000	1165(94)
	≥ 4000	75(6)

^a^ Data of three participants regarding obstetrical variables were missing

**Fig. 2:**
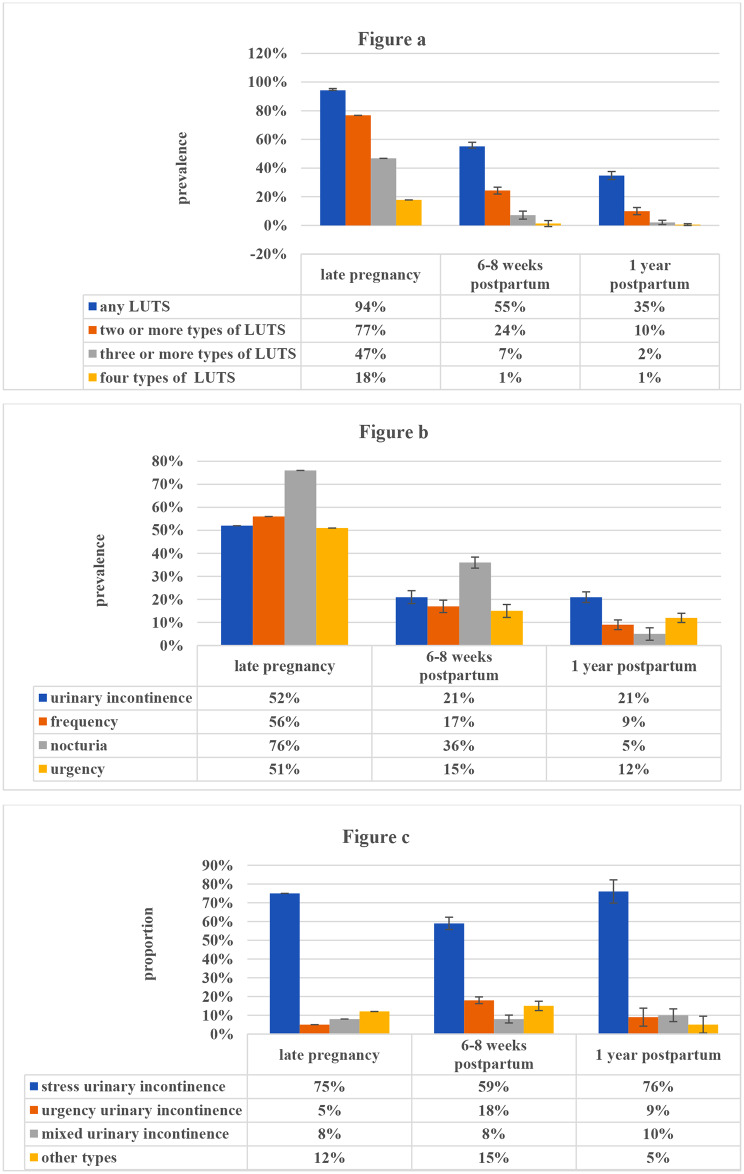
(**a**) The prevalence of overall LUTS during pregnancy and after childbirth; (**b**) The prevalence of individual LUTS during pregnancy and after childbirth; (**c**) The proportion of different types of urinary incontinence during pregnancy and after childbirth

The prevalence of LUTS was shown in Fig. 2. In late pregnancy, 94% of pregnant women had at least one type of storage symptoms and nearly half of the participants had three or more types of storage symptoms. Nocturia was the most prevalent LUTS in late pregnancy. At 6 to 8 weeks postpartum, more than half of the participants had at least one type of storage symptoms and nocturia was still the most prevalent LUTS. At one year postpartum, about one third of the participants had at least one type of storage symptoms and urinary incontinence was the most prevalent LUTS, followed by urgency, increased daytime frequency and nocturia. Overall, the prevalence of LUTS was highest in late pregnancy and declined significantly after childbirth. However, the prevalence of UI remained at 21% within one year after childbirth, amongst which stress urinary incontinence was the most common type.

As shown in Table 2, the majority of the participants leaked a small amount of urinary incontinence during pregnant and postnatal period. In late pregnancy, nearly half of the participants urinate three times or more at night. However, only 1% of the participants urinate three times or more at night at one year postpartum.

**Table 2:** The frequency and/or volume of lower urinary tract symptoms during pregnancy and after childbirth

Variables	Group	Late pregnancy n = 1243	6–8 weeks postpartum n = 1186	One year postpartum n = 1110
UI frequency	Never	597(48)	936(79)	875(79)
	About once a week or less often	432(35)	193(16)	210(19)
	Two or three times a week	91(7)	29(2)	13(1)
	About once a day	61(5)	17(2)	7(0.6)
	Several times a day	51(4)	9(0.8)	3(0.2)
	All the time	11(1)	2(0.2)	2(0.2)
UI volume	A small amount	633(98)	236(94)	229(97)
	A moderate amount	12(2)	14(6)	6(3)
	A large amount	1(0.1)	0(0)	0(0)
Daytime urination frequency ^a^	Seven times or less often	550(44)	989(83)	1005(90.5)
	Eight to fourteen times	628(51)	188(16)	100(9)
	Fifteen or more times	64(5)	9(1)	5(0.5)
Nocturnal urination frequency ^b^	Never	28(2)	228(19)	700(63)
	One time	264(21)	533(45)	356(32)
	Two times	429(35)	328(28)	46(4)
	Three or more times	519(42)	97(8)	8(1)
Urgency frequency ^a^	Never	606(49)	1014(85)	977(88)
	Less than once a week	210(17)	102(9)	96(8.6)
	One or more times a week	143(11)	47(4)	22(2)
	About one time a day	134(11)	10(1)	10(0.9)
	Two to four times a day	121(10)	10(0.8)	5(0.5)
	Five or more times a day	28(2)	3(0.2)	0(0)

^a^ Data of one participant regarding daytime urination frequency and urgency frequency during pregnancy were missing. ^b^ Data of three participants regarding nocturnal urination frequency during pregnancy were missing. UI: urinary incontinence

As shown in Table 3, ten candidate risk factors for urinary incontinence were included for multivariate analysis. Logistic regression analysis indicated that pre-pregnancy BMI, age at first birth, gestational diabetes mellitus and previous history of urinary incontinence were independent risk factors for urinary incontinence one year postpartum. Pre-pregnancy BMI, age at first birth, birth mode and previous history of urinary incontinence were independent predictors of stress urinary incontinence. Amongst the risk factors, urinary incontinence during pregnancy was the strongest predictor. All univariate analyses were shown in supplemental Tables 1, 2, 3, 4 and 5.

**Table 3:** Logistic regression analysis of risk factors for urinary incontinence one year postpartum

Variables	UI^a^		SUI^b^	
	OR(95%CI)	*p*	OR(95%CI)	*p*
Constant	—	<0.001	—	<0.001
Pre-pregnancy BMI	1.073(1.014–1.135)	0.014	1.067(1.003–1.134)	0.039
Age at first birth				
≤ 30	Reference group		Reference group	
>30	1.677(1.190–2.363)	0.003	1.679(1.156–2.438)	0.006
Gestational diabetes mellitus				
No	Reference group		—	
Yes	1.468(1.034–2.083)	0.032		
UI before pregnancy				
No	Reference group		Reference group	
Yes	2.293(1.515–3.471)	<0.001	2.289(1.476–3.552)	<0.001
UI during pregnancy				
No	Reference group		Reference group	
Yes	2.624(1.877–3.669)	<0.001	2.418(1.663–3.517)	<0.001
UI 6–8 weeks postpartum				
No	Reference group		Reference group	
Yes	2.011(1.406–2.874)	<0.001	1.797(1.224–2.637)	0.003
Birth mode				
Cesarean section	Reference group		Reference group	
Vaginal delivery	1.350(0.953–1.912)	0.091	1.664(1.125–2.460)	0.011

^a^Adjusted for the following variables: age at first birth, family history of UI, history of urinary tract infection, gestational diabetes mellitus, pre-pregnancy BMI, UI before pregnancy, UI during pregnancy, UI 6–8 weeks postpartum, birth mode and birth weight

^b^Adjusted for the following variables: age at first birth, family history of UI, history of constipation, gestational diabetes mellitus, pre-pregnancy BMI, UI before pregnancy, UI during pregnancy, UI 6–8 weeks postpartum, birth mode and birth weight

UI: urinary incontinence

As shown in Table 4, eight candidate risk factors for increased daytime frequency were identified for multivariate analysis. Logistic regression analysis indicated that pre-pregnancy BMI, increased daytime frequency during pregnancy and 6 to 8 weeks postpartum were independent predictors of increased daytime frequency one year postpartum. Women with a history of increased daytime frequency were more susceptible to suffer from increased daytime frequency one year postpartum (OR = 3.940, 95%CI = 2.530–6.137, *p*<0.001; OR = 2.032, 95%CI = 1.259–3.277, *p* = 0.004). However, pre-pregnancy BMI could protect women from suffering from increased daytime frequency (OR = 0.916, 95%CI = 0.842–0.996, *p* = 0.040).

**Table 4:** Logistic regression analysis of risk factors for increased daytime frequency one year postpartum

Variables	OR (95%CI)	*p*
Constant	—	0.139
Pre-pregnancy BMI	0.916(0.842–0.996)	0.040
Increased daytime frequency during pregnancy		
No	Reference group	
Yes	2.032(1.259–3.277)	0.004
Increased daytime frequency 6–8 weeks postpartum		
No	Reference group	
Yes	3.940(2.530–6.137)	<0.001
Age at first birth		
≤ 30	Reference group	
>30	1.494(0.947–2.355)	0.084

Adjusted for the following variables: pre-pregnancy BMI, tea consumption, fluid consumption, history of urinary tract infection, increased daytime frequency during pregnancy, increased daytime frequency 6–8 weeks postpartum, age at first birth and birth weight

As shown in Table 5, seven variables were included for multivariate analysis. Logistic regression analysis indicated that women older than 35 years, engaging in manual work, having a history of urinary tract infection and nocturia were more likely to suffer from nocturia one year postpartum. A history of nocturia 6 to 8 weeks postpartum was the strongest predictor of nocturia one year postpartum (OR = 7.273, 95%CI = 3.582–14.765, *p*<0.001), followed by nocturia during pregnancy and manual work (OR = 5.833, 95%CI = 1.386–24.537, *p* = 0.016; OR = 2.406, 95%CI = 1.204–4.807, *p* = 0.013).

**Table 5:** Logistic regression analysis of risk factors for nocturia one year postpartum

Variables	OR (95%CI)	*p*
Constant	—	<0.001
Age		
≤ 35	Reference group	
>35	2.159(1.079–4.320)	0.030
Job		
Mental labor	Reference group	
Manual labor	2.406(1.204–4.807)	0.013
History of urinary tract infection	2.286(1.197–4.364)	0.012
Nocturia during pregnancy	5.833(1.386–24.537)	0.016
Nocturia 6–8 weeks postpartum	7.273(3.582–14.765)	<0.001

Adjusted for the following variables: age, job, childhood enuresis, history of urinary tract infection, gestational diabetes mellitus, nocturia during pregnancy and nocturia 6–8 weeks postpartum

As shown in Table 6, four variables were included for multivariate analysis. Finally, urgency during pregnancy and 6 to 8 weeks postpartum were found to be predictors of urgency one year postpartum (OR = 2.534, 95%CI = 1.679–3.824, *p*<0.001; OR = 3.207, 95%CI = 2.112–4.871, *p*<0.001).

**Table 6:** Logistic regression analysis of risk factors for urgency one year postpartum

Variables	OR (95%CI)	*p*
Constant	—	<0.001
Urgency during pregnancy		
No	Reference group	
Yes	2.534(1.679–3.824)	<0.001
Urgency 6–8 weeks postpartum		
No	Reference group	
Yes	3.207(2.112–4.871)	<0.001

Adjusted for the following variables: coffee consumption, pre-pregnancy BMI, urgency during pregnancy and urgency 6–8 weeks postpartum

## Discussion

This study enrolled primiparas and multiparas and systematically explored the natural history of LUTS including urinary incontinence, increased daytime frequency, nocturia and urgency from late pregnancy to one year postpartum and associated risk factors for LUTS one year postpartum through a large prospective design, which could help health care providers get a novel and deep understanding of LUTS around childbirth and facilitate the early prevention of LUTS.

In the study, LUTS were highly prevalent in late pregnancy, which was similar to the results in the studies of pregnant women but much more common than that in general adult women [8, 11, 24, 25]. Among LUTS, nocturia was the most prevalent in late pregnancy, which was in consistent with the results of general adult women [8, 11]. However, studies regarding the incidence of nocturia remains sparse, especially in perinatal women [5]. The prevalence of all types of LUTS were higher than that of previous studies regarding pregnant women, which could be explained by the difference of included population in different studies [23, 24]. The prevalence of urinary incontinence in late pregnancy was 52%, of which stress urinary incontinence was more prevalent than other two types, which was in accordance with the results in previous studies [21, 37]. The prevalence of urinary incontinence during pregnancy was higher than that of another study conducted in China more than a decade ago, even in primiparous women with no history of incontinence, which needs further studies [22]. In addition, we found that urinary leakage around childbirth was a small amount in most participants. A recent systematic review also indicated that the majority of women suffered from mild to moderate urinary incontinence during pregnancy [38]. However, more attention should be paid to this population considering the high prevalence and its persistent effect in later life.

The prevalence of LUTS decreased significantly after childbirth, amongst which the prevalence of nocturia, increased daytime frequency and urgency decreased continuously within one year postpartum, while the prevalence of urinary incontinence remained unchanged after 6 to 8 weeks postpartum, indicating that the prevalence of urinary incontinence was more stable than that of other LUTS within one year postpartum. Similarly, Chan et al. found that the prevalence of urinary incontinence in primiparas was 23-28% within one year postpartum [30]. Besides, a systematic review showed that the pooled prevalence of urinary incontinence 3 months postpartum was 33% and small changes was found over time at one year postpartum [36]. This study implied that 6 to 8 weeks postpartum was an important period among the natural process of LUTS. At 6 to 8 weeks postpartum, pelvic organs returned to their original positions and interventions should be targeted [19]. To date, there is a lack of epidemiological studies exploring the natural process of all storage symptoms around childbirth. The results showed that about one third of women were affected by at least one type of LUTS at one year postpartum. Both health care providers and perinatal women should pay more attention to the risk assessment and prevention of LUTS during postnatal routine examination, facilitating the prevention of LUTS postpartum.

The etiology of LUTS was complex and uncertain. This study demonstrated that sociodemographic factors (age, job), obesity (BMI before pregnancy), medical history (gestational diabetes mellitus, urinary tract infection history, previous history of LUTS), and obstetrical factors (age at first birth, birth mode) were significant predictors of LUTS postpartum. We found that sociodemographic factors including age and job were independent predictors of nocturia one year postpartum. A nested case-control study conducted in general adult population showed that the prevalence of nocturia increased significantly with age [39]. In addition, we found that women engaging in manual work showed higher risk of suffering from nocturia one year postpartum, indicating that women with lower socioeconomic status were more likely to suffer from nocturia. Manual work may cause more pressure and injury to the pelvic floor of women, leading to the occurrence of nocturia. There is evidence that nocturia was associated with increased risks of falls, fractures and depression, and decreased quality of life [1, 5]. However, risk factors for nocturia were not well understood and most epidemiological studies were cross-sectional and focused on nonpregnant women [5]. Hence, more prospective studies are warranted. Strategies regarding the management of nocturia should be given to pregnant women aged more than 35 years and engaged in manual work as early as possible.

Obesity was a well-accepted risk factor for urinary incontinence [40, 41]. In the study, BMI before pregnancy was related to increased risk of urinary incontinence one year postpartum. Similarly, Schytt et al. found that women with BMI equal to or greater than 30 before pregnancy were more likely to suffer from urinary incontinence one year postpartum compared with women with normal weight [31]. A higher BMI may lead to increased pressure to the pelvic floor muscle and bladder, which may contribute to increased urethral mobility and urinary incontinence later in life [42]. The latest guideline of NICE showed that compared with weight gain during pregnancy, obesity before pregnancy had a greater impact on the occurrence of urinary incontinence [28]. Hence, more efforts should be made in weight management before pregnancy in order to decrease the risk of urinary incontinence postpartum. On the contrary, BMI before pregnancy was a protective factor for urinary frequency. However, a study enrolling community-dwelling nonpregnant women showed that women with greater BMI had greater risk of developing urinary frequency [43]. The inconsistency might be associated with different populations in different studies. In the above study, the average BMI of community-dwelling women was 27 to 28 and about 30% of the participants had BMI equal to or greater than 30, which was much greater than that in our study (M = 21). To date, little attention has been paid to explore the risk factors for urinary frequency in women, highlighting the need for more efforts in the epidemiological studies of urinary frequency.

We found that gestational diabetes mellitus and urinary tract infection history were predictors of urinary incontinence and nocturia postpartum, respectively. Our finding implied that gestational diabetes mellitus had a long-lasting impact on bladder health although it usually recovered after childbirth. Chuang et al. found that urinary incontinence was more prevalent and severe in women with gestational diabetes mellitus at two years postpartum [44]. The mechanism underlying the occurrence of urinary incontinence related to gestational diabetes mellitus was unclear. There is evidence that gestational diabetes mellitus may cause polyuria and bladder detrusor instability [42]. Besides, gestational diabetes mellitus was associated with alterations of pelvic floor muscle function during pregnancy such as decreased contractility, distensibility, or mobility, which may lead to long-term urinary incontinence postpartum [45]. In the study, women with a history of urinary tract infection were more susceptible to suffer from nocturia postpartum, which was in line with the result of a study conducted in general adult women [43] Health care providers should pay more attention to the lifestyle-related health education, postnatal follow-up and assessment of LUTS in women with gestational diabetes mellitus and urinary tract infection history, which would be beneficial for the prevention of LUTS postpartum. Previous history of LUTS played an important role in the development of LUTS postpartum. Among all the storage symptoms, previous history of LUTS was the strongest predictor of LUTS one year postpartum, which was in consistent with previous findings regarding urinary incontinence [17, 22, 46, 47]. Our findings implied that women with previous history of LUTS were at high risk of developing long-term LUTS. As a result, effective preventive strategies such as pelvic floor muscle training should be targeted timely.

Among the obstetrical factors, age at first birth was found to be the predictor of urinary incontinence postpartum, which was in consistent with previous studies [48–50]. Older age at first birth was associated with increased risk of developing long-term urinary incontinence postpartum and subsequent surgery in relation to stress urinary incontinence [48, 49]. However, there is evidence that age at first birth was not associated with urinary incontinence postpartum and the effect of maternal age at first birth was significant in younger women but attenuated with aging [51, 52]. Our finding could be explained by the evidence that advanced age at first birth seemed associated with increased possibilities of pelvic floor trauma, contributing to the occurrence of incontinence postpartum [53]. So far, studies regarding age at first birth in relation to urinary incontinence postpartum, particularly around childbirth, was sparse. Pregnant women aged more than 30 years at first birth should be informed the risk of developing incontinence postpartum and provided with effective preventive strategies during pregnant and postnatal period to prevent incontinence postpartum. Vaginal delivery was an independent predictor of stress urinary incontinence one year postpartum, which was consistent with previous studies [5]. However, no correlation with unspecified urinary incontinence was found indicating that the mechanisms underlying the occurrence of different types of urinary incontinence postpartum were different. Although vaginal delivery was associated with increased risk of developing incontinence postpartum, the risks and benefits of different birth modes should be balanced comprehensively from the perspective of the mother and baby in consultation about birth mode.

The study showed that lifestyle factors were not independent predictors of urinary incontinence one year postpartum. To date, the association between most lifestyle factors and LUTS remains inconclusive [54]. There are recommendations of lifestyle interventions (e.g., advise women with urinary incontinence to modify fluid intake and reduce caffeine intake) for women with LUTS, however, the quality of evidence was very low to low [28]. Therefore, more high-quality evidence is needed to inform decision making in the lifestyle intervention of LUTS.

## Limitations

This study has limitations. First, the study was conducted in a tertiary hospital, which may limit the generalizability of the results. Second, although this was a prospective study, there was potential recall bias regarding some variables such as urinary tract infection history and childhood enuresis.

## Conclusion

LUTS including urinary incontinence, increased daytime frequency, nocturia and urgency were highly prevalent around childbirth. The prevalence of nocturia, increased daytime frequency and urgency decreased continuously within one year postpartum, while the prevalence of urinary incontinence was more stable than that of other LUTS within one year postpartum. Sociodemographic factors (age, job), obesity (BMI before pregnancy), medical history (gestational diabetes mellitus, urinary tract infection history and previous history of LUTS) and obstetrical factors (age at first birth and birth mode) were significant predictors of LUTS postpartum. The findings could help health care providers get a comprehensive and deep insight of the natural process of LUTS and related risk factors in this population so that preventive interventions could be targeted in pregestational, pregnant and postnatal period.

### Electronic supplementary material

Below is the link to the electronic supplementary material.

Supplementary Material 1

## Data Availability

The datasets analyzed during the current study are available from the corresponding author upon reasonable request.

## Abbreviations

LUTS: Lower urinary tract symptoms
ICIQ-UI SF: International Consultation on Incontinence Modular Questionnaire-Urinary Incontinence Short Form
BMI: Body mass index
