# Supplementary material for: Epidemiological trends and risk factors related to lower urinary tract symptoms around childbirth: a one-year prospective study
Source: BMC Public Health. 2023 Oct 31;23:2134. doi: 10.1186/s12889-023-17065-w (PMC10617094; doi:10.1186/s12889-023-17065-w)
Supplement: Supplementary file 1 — Supplementary Material 1 [file 12889_2023_17065_MOESM1_ESM.docx]

Supplemental Table 1 Univariate analysis of risk factors for urinary incontinence one year postpartum

| Variables | group | Total sample  n（%）/M±SD | UI sample  n（%）/M±SD | *p* |
| --- | --- | --- | --- | --- |
| BMI before pregnancy（kg/m^2^） | — | 21.1±2.7 | 21.6±2.8 | 0.003 |
| Family history of UI | No | 1059（95） | 219（21） | 0.068 |
|  | Yes | 51（5） | 16（31） |  |
| History of urinary tract infection | No | 942（85） | 191（20） | 0.084 |
|  | Yes | 168（15） | 44（26） |  |
| UI before pregnancy | No | 980（88） | 180（18） | ＜0.001 |
|  | Yes | 130（12） | 55（42） |  |
| UI during pregnancy | No | 530（48） | 62（12） | ＜0.001 |
|  | Yes | 580（52） | 173（30） |  |
| UI 6-8 weeks postpartum | No | 874（79） | 150（17） | ＜0.001 |
|  | Yes | 236（21） | 85（36） |  |
| Gestational diabetes mellitus | No | 859（77） | 167（19） | 0.009 |
|  | Yes | 251（23） | 68（27） |  |
| Age at first birth （years） | ≤30 | 844（76） | 163（19） | 0.007 |
|  | ＞30 | 266（24） | 72（27） |  |
| Birth mode | Cesarean section | 454（41） | 80（18） | 0.016 |
|  | Vaginal delivery | 656（59） | 155（24） |  |
| Birth weight（g） | ＜4000 | 1041（94） | 216（21） | 0.181 |
|  | ≥4000 | 69（6） | 19（28） |  |

Supplemental Table 2 Univariate analysis of risk factors for stress urinary incontinence one year postpartum

| Variables | group | Total sample  n（%）/M±SD | UI sample  n（%）/M±SD | *p* |
| --- | --- | --- | --- | --- |
| BMI before pregnancy（kg/m^2^） | — | 21.1±2.7 | 21.5±2.5 | 0.033 |
| Family history of UI | No | 1059（95） | 165（16） | 0.060 |
|  | Yes | 51（5） | 13（8） |  |
| Constipation | No | 923（83） | 142（15） | 0.189 |
|  | Yes | 187（17） | 36（19） |  |
| UI before pregnancy | No | 980（88） | 135（14） | ＜0.001 |
|  | Yes | 130（12） | 43（33） |  |
| UI during pregnancy | No | 530（48） | 47（9） | ＜0.001 |
|  | Yes | 580（52） | 131（23） |  |
| UI 6-8 weeks postpartum | No | 874（79） | 112（13） | ＜0.001 |
|  | Yes | 236（21） | 66（28） |  |
| Gestational diabetes mellitus | No | 859（77） | 127（15） | 0.036 |
|  | Yes | 251（23） | 51（20） |  |
| Age at first birth （years） | ≤30 | 844（76） | 123（15） | 0.018 |
|  | ＞30 | 266（24） | 55（21） |  |
| Birth mode | Cesarean section | 454（41） | 54（12） | 0.002 |
|  | Vaginal delivery | 656（59） | 124（19） |  |
| Birth weight（g） | ＜4000 | 1041（94） | 163（16） | 0.182 |
|  | ≥4000 | 69（6） | 15（22） |  |

Supplemental Table 3 Univariate analysis of risk factors for increased daytime frequency one year postpartum

| Variables | group | Total sample  n（%）/M±SD | UI sample  n（%）/M±SD | *p* |
| --- | --- | --- | --- | --- |
| BMI before pregnancy（kg/m^2^） | — | 21.1±2.7 | 20.6±2.2 | 0.019 |
| Tea consumption | No | 1034（93） | 94（9） | 0.122 |
|  | Yes | 76（7） | 11（15） |  |
| Fluid consumption | No | 445（40） | 33（7） | 0.057 |
|  | Yes | 665（60） | 72（11） |  |
| History of urinary tract infection | No | 942（85） | 84（9） | 0.144 |
|  | Yes | 168（15） | 21（13） |  |
| Increased daytime frequency during pregnancy | No | 497（45） | 26（5） | ＜0.001 |
|  | Yes | 612（55） | 79（13） |  |
| Increased daytime frequency 6-8 weeks postpartum | No | 930（84） | 61（7） | ＜0.001 |
|  | Yes | 180（16） | 44（24） |  |
| Age at first birth （years） | ≤30 | 844（76） | 72（9） | 0.060 |
|  | ＞30 | 266（24） | 33（12） |  |
| Birth weight（g） | ＜4000 | 1041（94） | 102（10） | 0.134 |
|  | ≥4000 | 69（6） | 3（4） |  |

Supplemental Table 4 Univariate analysis of risk factors for nocturia one year postpartum

| Variables | group | Total sample  n（%） | UI sample  n（%） | *p* |
| --- | --- | --- | --- | --- |
| Age | ≤35 | 964（87） | 41（4） | 0.015 |
|  | ＞35 | 146（13） | 13（9） |  |
| Job | Mental labor | 975（88） | 41（4） | 0.006 |
|  | Manual labor | 135（12） | 13（10） |  |
| History of urinary tract infection | No | 942（85） | 38（4） | 0.002 |
|  | Yes | 168（15） | 16（10） |  |
| Childhood enuresis | No | 1009（91） | 46（5） | 0.134 |
|  | Yes | 101（9） | 8（8） |  |
| Gestational diabetes mellitus | No | 859（77） | 37（4） | 0.110 |
|  | Yes | 251（23） | 17（7） |  |
| Nocturia during pregnancy | No | 271（24） | 2（1） | ＜0.001 |
|  | Yes | 838（76） | 52（6） |  |
| Nocturia 6-8 weeks postpartum | No | 715（64） | 10（1） | ＜0.001 |
|  | Yes | 395（36） | 44（11） |  |

Supplemental Table 5 Univariate analysis of risk factors for urgency one year postpartum

| Variables | group | Total sample  n（%）/M±SD | UI sample  n（%）/M±SD | *p* |
| --- | --- | --- | --- | --- |
| BMI before pregnancy（kg/m^2^） | — | 21.1±2.7 | 21.4±2.9 | 0.191 |
| Coffee consumption | No | 1039（94） | 128（12） | 0.185 |
|  | Yes | 71（6） | 5（7） |  |
| Urgency during pregnancy | No | 547（49） | 36（7） | ＜0.001 |
|  | Yes | 562（51） | 97（17） |  |
| Urgency 6-8 weeks postpartum | No | 950（86） | 88（9） | ＜0.001 |
|  | Yes | 160（14） | 45（28） |  |
